# Predictive value of hyperglycemia on infection in critically ill patients with acute pancreatitis

**DOI:** 10.1038/s41598-023-30608-w

**Published:** 2023-03-13

**Authors:** Yingzhi Jin, Shaoyu Tao, Guang Yu, Chengyi Li, Zanqun Hu, Longjian Jiang

**Affiliations:** 1 Department of Emergency and Critical Care Medicine, The Second Affiliated Hospital of Nanchang University, No.1 Minde Road, Nanchang, 330006 Jiangxi Province China; 2 Department of Clinical Medicine, The Second Clinical Medical College, Nanchang University, Nanchang, China

**Keywords:** Gastroenterology, Infectious diseases

## Abstract

To analyze the predictive value of hyperglycemia on the extrapancreatic infection (EPI) and infected pancreatic necrosis (IPN) of severe patients with acute pancreatitis (AP). We enrolled 234 patients with acute pancreatitis admitted to the intensive care unit (ICU) of the Second Affiliated Hospital of Nanchang University from July 2017 to July 2022 for a retrospective cohort study. We collected maximum blood glucose values three times after admission to the ICU within 120 h (Glu1: 0–24 h, Glu2: 24–48 h, Glu3: 48–120 h), the levels of leucocyte, blood urea nitrogen (BUN), C-reactive protein (CRP), procalcitonin (PCT), and albumin within 24 h after admission to the ICU, and the BISAP and SIRS scores of all patients within 24 h. EPI was taken as the primary outcome indicator and IPN as the secondary outcome indicator. The accuracy of blood glucose values in predicting acute pancreatitis infection was measured by the area under the curve (AUC). A total of 56 patients appeared EPI. Univariate analysis showed that Glu3 was associated with IPN in critically ill patients with AP. Multivariate logistic regression analysis showed that Glu2, Glu3, and SIRS > 48 h were associated with EPI in critically ill patients with AP. The AUCs of Glu2 and Glu3 to predict EPI were 0.805(95%CI: 0.717–0.892) and 0.782(95%CI: 0.685–0.878), respectively, and the cutoff values were 12.60 mmol/L and 14.75 mmol/L, respectively. The AUC of Glu2 combined with Glu3 to predict EPI was 0.812(0.725–0.899). The maximum blood glucose on Day2-5 after admission to the ICU can predict infection in critically ill patients with AP. There are differences in etiology while glucose predicting infection. Patients with hypertriglyceridemia AP need to intervene blood glucose levels more actively and earlier, and control it more strictly.

## Introduction

Although acute pancreatitis (AP) is a chemical inflammation, it is susceptible to infection in the course of the disease^1^, and when it happens, the mortality rate will increase^2^. The time to onset of infection varies from days to weeks, and common types of infection are extrapancreatic infection (EPI), abdominal infection, and infected (peri)pancreatic necrosis (IPN). EPI usually happens in the early phase, including pulmonary infection, bacteriemia, urinary tract infection, and catheter infection, and is one of the important reasons for disease aggravation and even death^3^.

IPN is a serious complication of AP, about 30% of patients with necrotizing pancreatitis develop a secondary infection, and the mortality reaches 30%^4^. However, prophylactic use of antibiotics does not reduce the incidence of IPN, instead increases the risk of multidrug-resistant and fungal infections^5^.Therefore, in the early course of the disease, it is particularly important to identify whether the patient has a potential infection risk and to intervene at the best time. SOFA, SIRS, Ranson's, BISAP, and MMS had medium performance in predicting IPN in HTG-AP^6^. On the univariate analysis of the risk factors for developing EPI, there is a relationship between EPI development and leukocyte level, APACHE-II > 8, serum calcium, BUN level, renal failure, and persistent SIRS at the first week^7^. However, neither various scoring systems nor inflammatory nor infectious indicators can accurately predict in time. Stress hyperglycemia can increase the risk of infection, and studies have shown that blood glucose-related indicators are associated with the prognosis of AP patients^8–10^, one of which mentioned that the maximum blood glucose has the highest accuracy in predicting death. In clinical studies, no one has explored the correlation between early blood glucose level and EPI and IPN in AP patients, and whether it can be used as an early predictor of them and the level of blood glucose control.

Based on previous studies, this study selected the maximum blood glucose as an exposure factor and aimed to analyze its predictive value on the occurrence of EPI and IPN in critically ill patients with AP to intervene in the blood glucose level at an appropriate time to improve the prognosis of patients.

## Materials and methods

The study was reviewed and approved by the Second Affiliated Hospital of Nanchang University medical research ethics committee (No. 037) with an exemption of consent for available anonymized clinical data analyzed in this study. All methods and intervene were performed in accordance with relevant guidelines and regulations.

The clinical data of 234 AP patients admitted to the ICU in the Second Affiliated Hospital of Nanchang University in recent 5 years were retrospectively analyzed. Patients who met any one of the exclusion criteria were excluded in the study: (1) age < 18 years old, or > 80 years old, or pregnancy; (2) extreme blood glucose values; (3) discharged without medical advice; (4) missing critical data; (5) patients transferred to our hospital more than 4 days after the onset of abdominal pain; (6) primary diagnosis was not AP.

We collected data by querying patients’ electronic medical records including general patient information, such as gender, age, etiology, and pre-existing diabetes; main variables: the maximum blood glucose level (Glu1) within 24 h, the maximum blood glucose level (Glu2) within 24–48 h, and the maximum blood glucose level within 48–120 h (Glu3) of patients after admission to the ICU; imaging data: CT, DR, MRCP; laboratory data: leucocyte, blood urea nitrogen (BUN), C-reactive protein (CRP), procalcitonin (PCT), albumin levels within 24 h after admission to the ICU, glycosylated hemoglobin, pathogenic microorganism culture results. BISAP and SIRS scores were calculated using the worst laboratory parameters and vital signs within 24 h after admission. The primary outcome was EPI and the secondary outcome was IPN.

## Definition

We included 234 patients with AP according to the 2012 Atlanta definition^11^. The diagnosis of AP requires two of the following three features: (1) abdominal pain consistent with acute pancreatitis (acute onset of a persistent, severe, epigastric pain often radiating to the back); (2) serum lipase activity (or amylase activity) at least three times greater than the upper limit of normal; and (3) characteristic findings of acute pancreatitis on contrast-enhanced computed tomography (CECT) and less commonly magnetic resonance imaging (MRI) or transabdominal ultrasonography.

According to its criteria, AP is divided into three degrees of severity: mild (MAP), moderately severe (MSAP), and severe (SAP). MAP is characterized by the absence of organ failure and the absence of local or systemic complications, whereas SAP is defined by the presence of persistent organ failure (≥ 48 h); MSAP includes the presence of transient organ failure (< 48 h) or local or systemic complications without persistent organ failure.

The maximum blood glucose value was defined as the highest value of all blood glucose values monitored every two hours after the patient was admitted to the ICU.

Infected (peri)pancreatic necrosis (IPN) includes infection of early acute necrotic collection (ANC ≤ 4 weeks) and of late walled-off necrosis (WON > 4 weeks). The diagnosis of infection can combine with the patient’s clinical manifestations (abdominal pain, fever, oliguria, hemodynamic instability) or by the presence of gas within the collection seen on CECT, or when the first puncture of percutaneous drainage is positive for bacteria and/or fungi on Gram stain and/or culture^11^.

Extrapancreatic infection (EPI) was defined as a new pulmonary infection, bacteremia, urinary tract infection, and catheter-related bloodstream infection. The diagnosis is based on a combination of sputum culture, blood culture, urinalysis, catheter tip culture, chest and abdomen CT, and patient clinical manifestations and signs.

## Statistical analysis

SPSS 26.0 version was used for analysis. We performed a normal distribution test on the quantitative data and found that the normality was not satisfied except for age. For convenience, a nonparametric test was used for univariate analysis. A chi-square test was used for qualitative data. *P* < 0.05 was accepted as statistically significant. The above-mentioned statistically significant variables were included in binary logistic regression for multivariate analysis to identify risk factors for EPI and IPN. The receiver operating characteristic curve (ROC curve) was drawn, and the predictive efficacy was analyzed by the area under the curve (AUC), and the cut-off value was found by the Youden index. Medcalc software was used to analyze whether the difference between each ROC curve was statistically significant. The total percentage of missing data was small. No special treatment for missing variables was considered.

## Results

**Figure 1:**
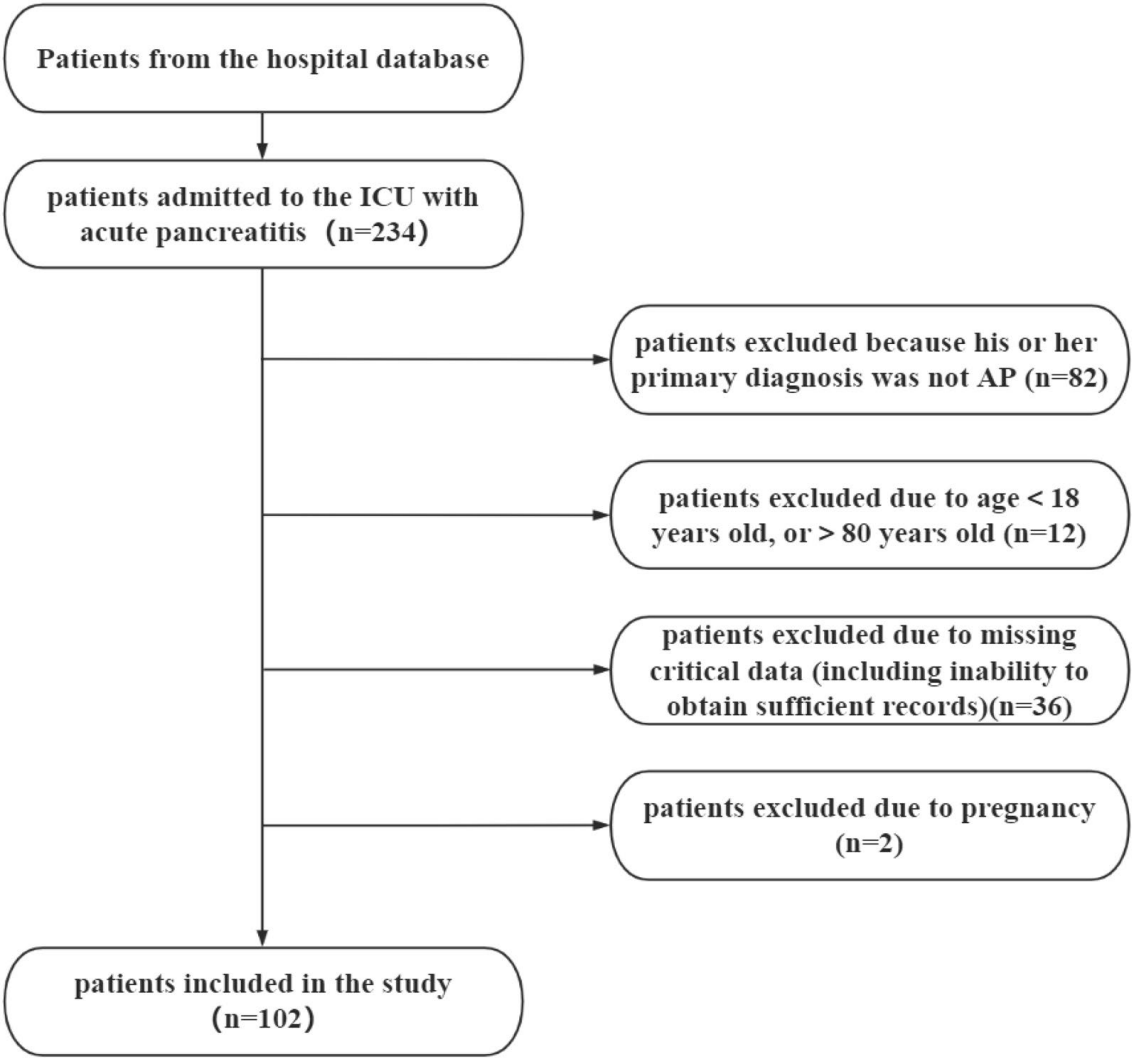
Flow chart of the study selection process.

Among 234 patients who presented to the ICU with acute pancreatitis between June 2017 and June 2022, 132 were excluded. A total of 102 patients were retrospectively analyzed (Fig. 1).

### Patient Characteristics

Table 1 shows the characteristics of the study population. The median age of the study population was 49.5 years, and 34 of them (33%) were female. The etiology was gallstone in 29 patients and hyperlipidemia in 42 patients. Ten patients (9.9%) were diagnosed as MAP, 31(30.7%) were MSAP and 60 (58.8%) were SAP. There were 33 patients with pre-existing diabetes. EPI occurred in 56 (54%) patients, including 31 new pulmonary infections, 32 bacteremia, 6 catheter-related bloodstream infections, and 2 urinary tract infections. IPN occurred in 18 patients. Seventy patients developed persistent organ failure and fourteen patients died during hospitalization. Compare the dynamic changes in blood glucose during the course of AP. We found that the maximum blood glucose value increases over time.

**Table 1:** Baseline characteristics and overall outcomes of study populations.

	n	Median (P_25_, P_75_) or (%)
Age, year	102	49.5 (40.0, 63.0)
Leucocyte, 10^9^/L	102	15.2 (11.3, 19.2)
BUN, mmol/L	102	9.5 (5.8, 16.4)
PCT, ng/ml	102	3.5 (1.0, 14.6)
ALB, g/L	101	30.8 (28.6, 35.0)
HbA1c, %	87	6.0 (5.5, 7.8)
Glu1, mmol/L	102	14.4 (11.0, 17.4)
Glu2, mmol/L	102	13.5 (10.8, 16.7)
Glu3, mmol/L	101	15.9 (12.3, 18.7)
BISAP, score	102	2.0 (1.0 ,2.0)
Time from onset to monitoring (days)	102	2.0 (1.0, 4.0)
Stay in ICU (days)	101	10.0 (5.0, 18.0)
Gender		
Male	68	67.0
Female	34	33.0
Severity		
MAP	10	9.9
MSAP	31	30.7
SAP	60	58.8
Etiology		
Gallstone	29	28.4
Hypertriglyceridemia	42	41.2
Others	31	30.4
Diabetes	33	32.4
CRP > 150 mg/L	70	74.5
SIRS at presentation	96	94.1
SIRS > 48 h	71	69.6
EPI	56	54.9
Pulmonary	31	
Bacteria	32	
Catheter-related	6	
Urinary tract	2	
Abdominal infection	39	38.2
IPN	18	17.6
Organ failure	70	68.6
Mortality	14	13.7

BUN: blood urea nitrogen; PCT: procalcitonin; ALB: albumin; HbA1c: glycosylated hemoglobin; Glu 1: maximum blood glucose value on day 1; Glu 2: maximum blood glucose value on day 2; Glu 3: maximum blood glucose value on day 3–5; BISAP: the bedside index for severity of acute pancreatitis; CRP: C-reactive protein; SIRS: systemic inflammatory response syndrome; EPI: extrapancreatic infection; IPN: infected pancreatic necrosis.

### Univariate analysis

Table 2 shows that patients older, with higher BUN, higher PCT levels within 24 h after ICU admission, persistent SIRS more than 48 h, and higher Glu2 and Glu3 levels were more likely to develop EPI. The differences were all statistically significant. However, leukocytes, albumin, CRP, HbA1c, and Glu1 were not the factors influencing the occurrence of EPI in critically ill patients with AP. Table 3 shows that patients with EPI are more prone to abdominal infection, organ failure and longer stay in ICU significantly.

**Table 2:** Comparison of baseline characteristics and clinical data in patients with and without EPI.

	EPI, no	EPI, yes	*P* value
	N = 46	N = 56	
Age, years	46.0(35.0,54.0)	52.5(44.0,67.3)	0.011
Leucocyte, 10^9^/L	15.7(11.6,19.1)	14.8(11.1,19.4)	0.794
BUN, mmol/L	6.8(4.5,10.2)	11.3(7.3,19.3)	0.001
PCT, ng/ml	1.5(0.5,8.3)	5.9(2.1,22.7)	0.001
ALB, g/L	31.0(28.7,34.4)	30.8(28.3,36.7)	0.859
HbA1c, %	6.2(5.5,8.2)	6.0(5.5,7.1)	0.749
Glu1, mmol/L	12.5(10.5,17.1)	14.8(11.9,17.7)	0.087
Glu2, mmol/L	11.0(9.8,13.8)	15.3(13.1,17.9)	0.001
Glu3, mmol/L	12.6(10.3,16.5)	17.2(15.5,19.1)	0.001
BISAP, score	2.0(1.0,2.0)	2.0(1.3,3.0)	0.012
Men	35(51.5%)	33(48.5%)	0.067
Etiology			0.234
Gallstone	12(41.4%)	17(58.6%)	
Hypertriglyceridemia	23(54.8%)	19(45.2%)	
Others	11(35.5%)	20(64.5%)	
Diabetes	19(57.6%)	14(42.4%)	0.08
CRP > 150 mg/L	30(42.9%)	40(57.1%)	0.800
SIRS at presentation	41(42.7%)	55(57.3%)	0.052
SIRS > 48 h	25(35.2%)	46(64.8%)	0.002

BUN: blood urea nitrogen; PCT: procalcitonin; ALB: albumin; HbA1c: glycosylated hemoglobin; Glu 1: maximum blood glucose value on day 1; Glu 2: maximum blood glucose value on day 2; Glu 3: maximum blood glucose value on day 3–5; BISAP: the bedside index for severity of acute pancreatitis; CRP: C-reactive protein; SIRS: systemic inflammatory response syndrome.

**Table 3:** Comparison of outcomes in patients with and without EPI.

	EPI, no	EPI, yes	*P* value
	N = 46	N = 56	
IPN	6 (13.0%)	12 (21.4%)	0.269
Abdominal infection	8 (17.4%)	31 (55.4%)	0.001
Organ failures	19 (41.3%)	51 (91.1%)	0.001
Stay in ICU (days)	5.00 (3.75,11.25)	12.00 (7.00,21.00)	0.001
In-ICU death	3 (6.5%)	11 (19.6%)	0.055

EPI: extrapancreatic infection; IPN: infected pancreatic necrosis; ICU: intensive care unit.

### Multivariate analysis

Indicators with significant differences between EPI and non-EPI (except blood glucose indicators) were used as adjustment variables for multivariate logistic regression analysis of Glu2 and Glu3. In multivariate analysis (Tables 4 and 5), Glu2 and Glu3 were independent risk factors for EPI in AP patients (*OR* = 1.402, 95%CI 1.183–1.660; *OR* = 1.353, 95%CI 1.152–1.590, respectively). Compared with transient SIRS, persistent SIRS for more than 48 h will increase the risk of EPI, and the difference was statistically significant. In the univariate analysis, only Glu3 is related to the occurrence of IPN, so multivariate analysis cannot be performed like EPI (Table 6).

**Table 4:** Binary logistic regression analysis of EPI risk factors in critically ill patients with AP during ICU stay 1.

	Adjusted OR(95%CI)	*P* value
Glu2,mmol/L	1.402 (1.183,1.660)	0.001
SIRS > 48 h	4.291 (1.368,13.466)	0.013

Glu 1: maximum blood glucose value on day 1; Glu 2: maximum blood glucose value on day 2; Glu 3: maximum blood glucose value on day 3–5.

**Table 5:** Binary logistic regression analysis of EPI risk factors in critically ill patients with AP during ICU stay 2.

	Adjusted OR(95%CI)	*P* value
Glu3, mmol/L	1.353 (1.152,1.590)	0.001
Age, year	1.050 (1.004,1.098)	0.033
SIRS > 48 h	3.811 (1.227,11.840)	0.021

Glu 1: maximum blood glucose value on day 1; Glu 2: maximum blood glucose value on day 2; Glu 3: maximum blood glucose value on day 3–5.

**Table 6:** Comparison of baseline characteristics and clinical data in patients with and without IPN.

	IPN, no	IPN, yes	*P* value
	N = 84	N = 18	
Age, years	49.5 (42.0,65.0)	45.0 (31.0,59.5)	0.115
Leucocyte, 10^9^/L	15.5 (11.3,19.1)	14.7 (11.1,20.3)	0.847
BUN, mmol/L	9.5 (5.8,16.1)	9.5 (5.4,18.4)	0.661
PCT, ng/ml	3.7 (1.0,15.8)	3.1 (1.8,11.3)	0.742
ALB, g/L	31.2 (28.7,34.1)	29.7 (27.6,39.5)	0.943
HbA1c, %	5.9(5.5,7.1)	6.3(5.6,10.2)	0.390
Glu1, mmol/L	13.9(11.0,17.1)	15.8(12.4,18.4)	0.257
Glu2, mmol/L	13.4(10.7,16.1)	14.9(12.3,17.8)	0.086
Glu3, mmol/L	15.3(12.3,18.3)	18.8(14.9,21.7)	0.011
BISAP, score	2.0(1.0,2.0)	2.0(2.0,3.0)	0.272
Men	55 (80.9%)	13 (19.1%)	0.582
Etiology			
Gallstone	24 (82.8%)	5 (17.2%)	0.639
Hypertriglyceridemia	33 (78.6%)	9 (21.4%)	
Others	27(87.1%)	4 (12.9%)	
Diabetes	27 (81.8%)	6 (18.2%)	0.922
CRP > 150 mg/L	57 (81.4%)	13 (18.6%)	1.000
SIRS at presentation	78 (81.3%)	18 (18.0%)	0.242
SIRS > 48 h	58 (81.7%)	13 (18.3%)	0.790

BUN: blood urea nitrogen; PCT: procalcitonin; ALB: albumin; HbA1c: glycosylated hemoglobin; Glu 1: maximum blood glucose value on day 1; Glu 2: maximum blood glucose value on day 2; Glu 3: maximum blood glucose value on day 3–5; BISAP: the bedside index for severity of acute pancreatitis; CRP: ﻿C-reactive protein; SIRS: systemic inflammatory response syndrome.

### The ability of blood glucose levels to predict EPI

**Figure 2:**
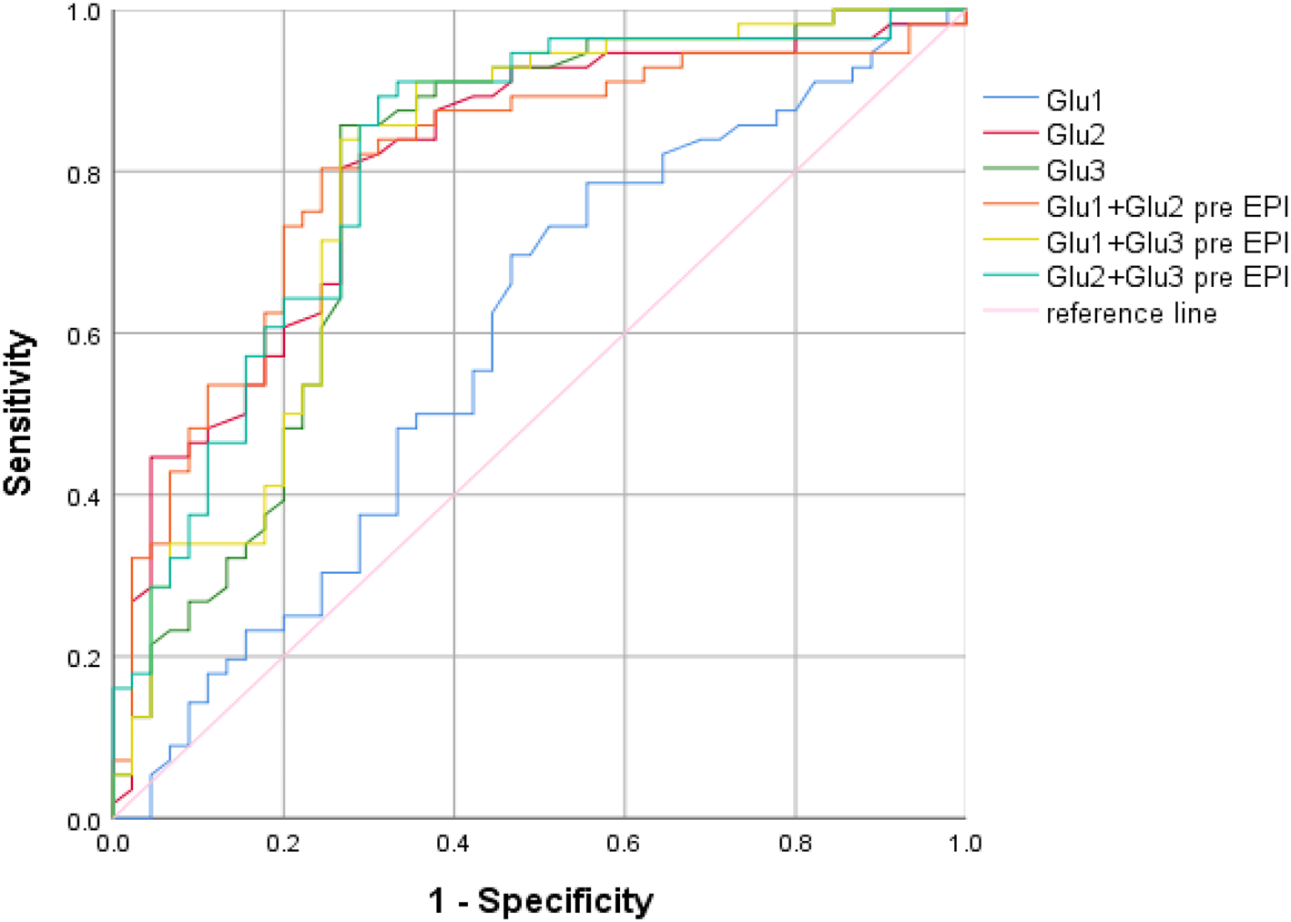
The ROC curve of maximum blood glucose at different periods alone or in combination to predict EPI in critically ill patients with AP.

The ROC curve was used to evaluate the predictive efficacy of maximum blood glucose at different times on EPI in critically ill patients with AP. According to Fig. 2 and Table 7, Glu1 had no predictive value for EPI. The predictive efficacy of Glu2 (AUC: 0.805) was higher than that of Glu3 (AUC: 0.782), and the cutoff values were 12.60 mmol/L and 14.75 mmol/L, respectively. Though the predictive power of individual values is not as good as that of the Glu2 + Glu3 (AUC: 0.812), the difference was not statistically significant.

**Table 7:** The predictive value of maximum blood glucose for EPI during ICU stay.

Cause	AUC	*P*	95%CI	Youd-en index	Cut-off Value (mmol/L)	Sensitivity (%)	Specificity (%)
Gallstone							
Glu1,mmol/L	0.435	0.553	0.218–0.653	0.139	11.00	0.722	0.417
Glu2,mmol/L	0.789	0.008	0.605–0.974	0.583	11.95	0.833	0.750
Glu3,mmol/L	0.873	0.001	0.746–0.999	0.694	14.73	0.778	0.917
Glu1 + Glu2	0.819	0.003	0.639–1.000	0.694	0.484	0.778	0.917
Glu1 + Glu3	0.870	0.001	0.738–1.000	0.694	0.510	0.778	0.917
Glu2 + Glu3	0.833	0.002	0.671–0.995	0.611	0.414	0.778	0.833
Hypertriglyceridemia							
Glu1,mmol/L	0.641	0.091	0.484–0.797	0.273	11.65	0.833	0.440
Glu2,mmol/L	0.822	0.001	0.704–0.941	0.597	12.25	0.917	0.680
Glu3,mmol/L	0.705	0.014	0.549–0.861	0.557	14.75	0.917	0.640
Glu1 + Glu2	0.822	0.001	0.703–0.941	0.595	0.399	0.875	0.720
Glu1 + Glu3	0.698	0.017	0.539–0.857	0.598	0.431	0.958	0.640
Glu2 + Glu3	0.823	0.001	0.706–0.940	0.557	0.330	0.917	0.640
All cause							
Glu1,mmol/L	0.593	0.111	0.479–0.706	0.230	11.50	0.786	0.444
Glu2,mmol/L	0.805	0.001	0.717–0.892	0.537	12.60	0.804	0.733
Glu3,mmol/L	0.782	0.001	0.685–0.878	0.590	14.75	0.857	0.733
Glu1 + Glu2	0.806	0.001	0.718–0.894	0.559	0.48	0.804	0.756
Glu1 + Glu3	0.793	0.001	0.700–0.886	0.572	0.51	0.839	0.733
Glu2 + Glu3	0.812	0.001	0.725–0.899	0.582	0.41	0.893	0.689

Glu 1: maximum blood glucose value on day 1; Glu 2: maximum blood glucose value on day 2; Glu 3: maximum blood glucose value on day 3–5.

### The ability of blood glucose levels to predict abdominal infection

**Figure 3:**
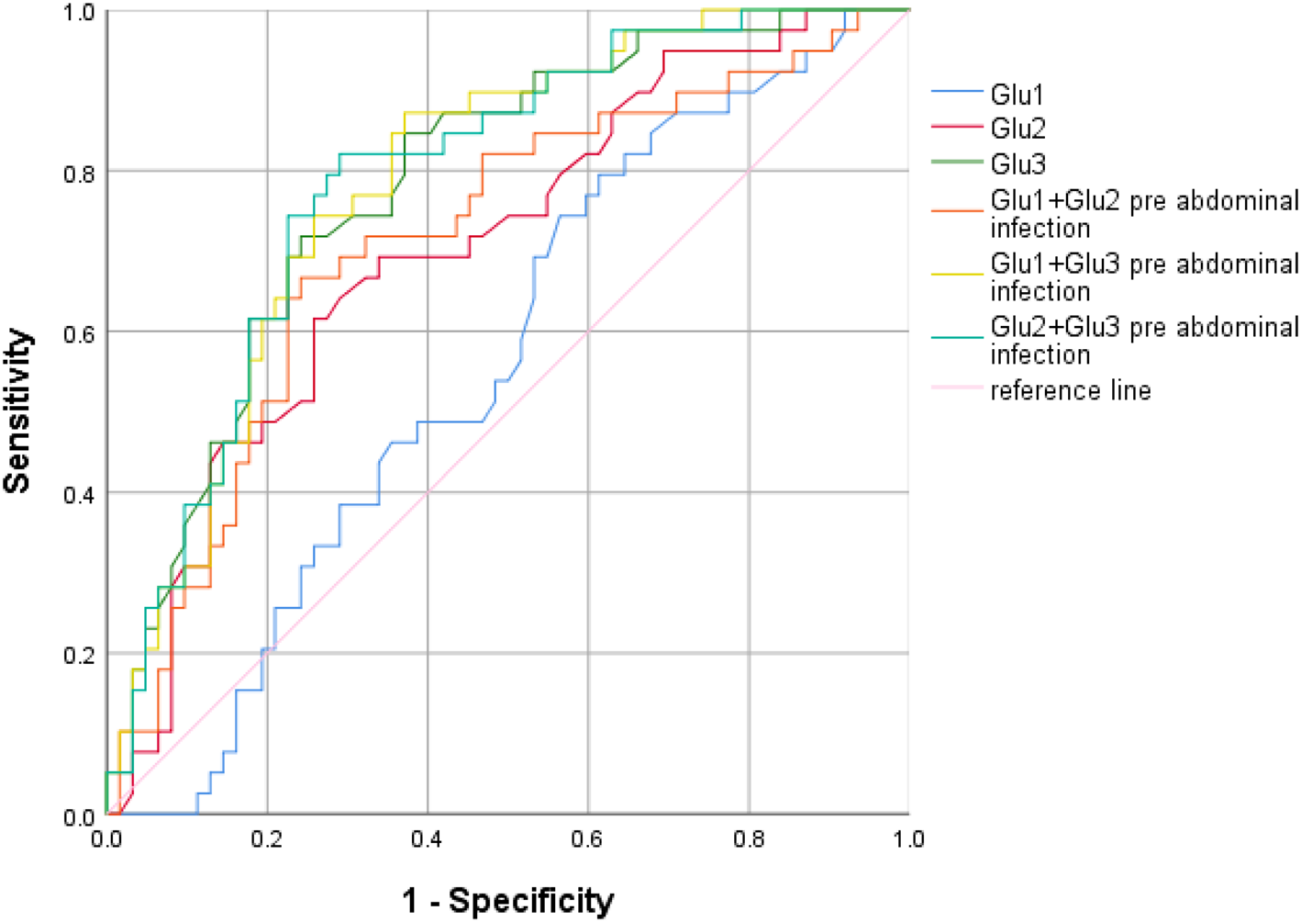
The ROC curve of maximum blood glucose at different periods alone or in combination to predict abdominal infection in critically ill patients with AP.

The ROC curve was used to evaluate the predictive efficacy of maximum blood glucose at different times on abdominal infection in critically ill patients with AP. According to Fig. 3 and Table 8, only Glu3 had the predictive value (AUC: 0.783, *P* = 0.046) and the cutoff value was 16.45 mmol/L. The AUCs of Glu1 + Glu3 and Glu 2 + Glu3 were 0.787 and 0.790, respectively.

**Table 8:** The predictive value of maximum blood glucose for abdominal infection during ICU stay.

Cause	AUC	*P*	95%CI	Youd-en index	Cut-off Value (mmol/L)	Sensitivity (%)	Specificity (%)
Gallstone							
Glu1,mmol/L	0.43	0.517	0.220–0.640	0.14	9.80	0.846	0.294
Glu2,mmol/L	0.597	0.368	0.378–0.816	0.303	14.35	0.538	0.765
Glu3,mmol/L	0.692	0.075	0.492–0.893	0.498	17.95	0.615	0.882
Glu1 + Glu2	0.629	0.233	0.414–0.844	0.362	0.395	0.538	0.824
Glu1 + Glu3	0.674	0.107	0.473–0.875	0.398	0.303	0.692	0.706
Glu2 + Glu3	0.688	0.082	0.487–0.889	0.498	0.489	0.615	0.882
Hypertriglyceridemia							
Glu1,mmol/L	0.659	0.084	0.508–0.811	0.386	12.45	0.929	0.457
Glu2,mmol/L	0.754	0.006	0.600–0.908	0.471	15.60	0.643	0.829
Glu3,mmol/L	0.844	0.001	0.730–0.958	0.571	16.60	0.857	0.714
Glu1 + Glu2	0.743	0.008	0.588–0.897	0.471	0.356	0.643	0.829
Glu1 + Glu3	0.839	0.001	0.726–0.952	0.600	0.309	0.857	0.743
Glu2 + Glu3	0.861	0.001	0.751–0.971	0.614	0.233	0.929	0.686
All cause							
Glu1,mmol/L	0.56	0.057	0.448–0.672	0.182	11.50	0.795	0.387
Glu2,mmol/L	0.703	0.053	0.600–0.807	0.357	14.65	0.615	0.742
Glu3,mmol/L	0.783	0.046	0.693–0.873	0.476	16.45	0.718	0.758
Glu1 + Glu2	0.714	0.053	0.609–0.818	0.425	0.39	0.667	0.758
Glu1 + Glu3	0.787	0.045	0.699–0.875	0.501	0.30	0.872	0.629
Glu2 + Glu3	0.79	0.045	0.701–0.879	0.530	0.38	0.821	0.710

Glu 1: maximum blood glucose value on day 1; Glu 2: maximum blood glucose value on day 2; Glu 3: maximum blood glucose value on day 3–5.

### The ability of blood glucose levels to predict IPN

**Figure 4:**
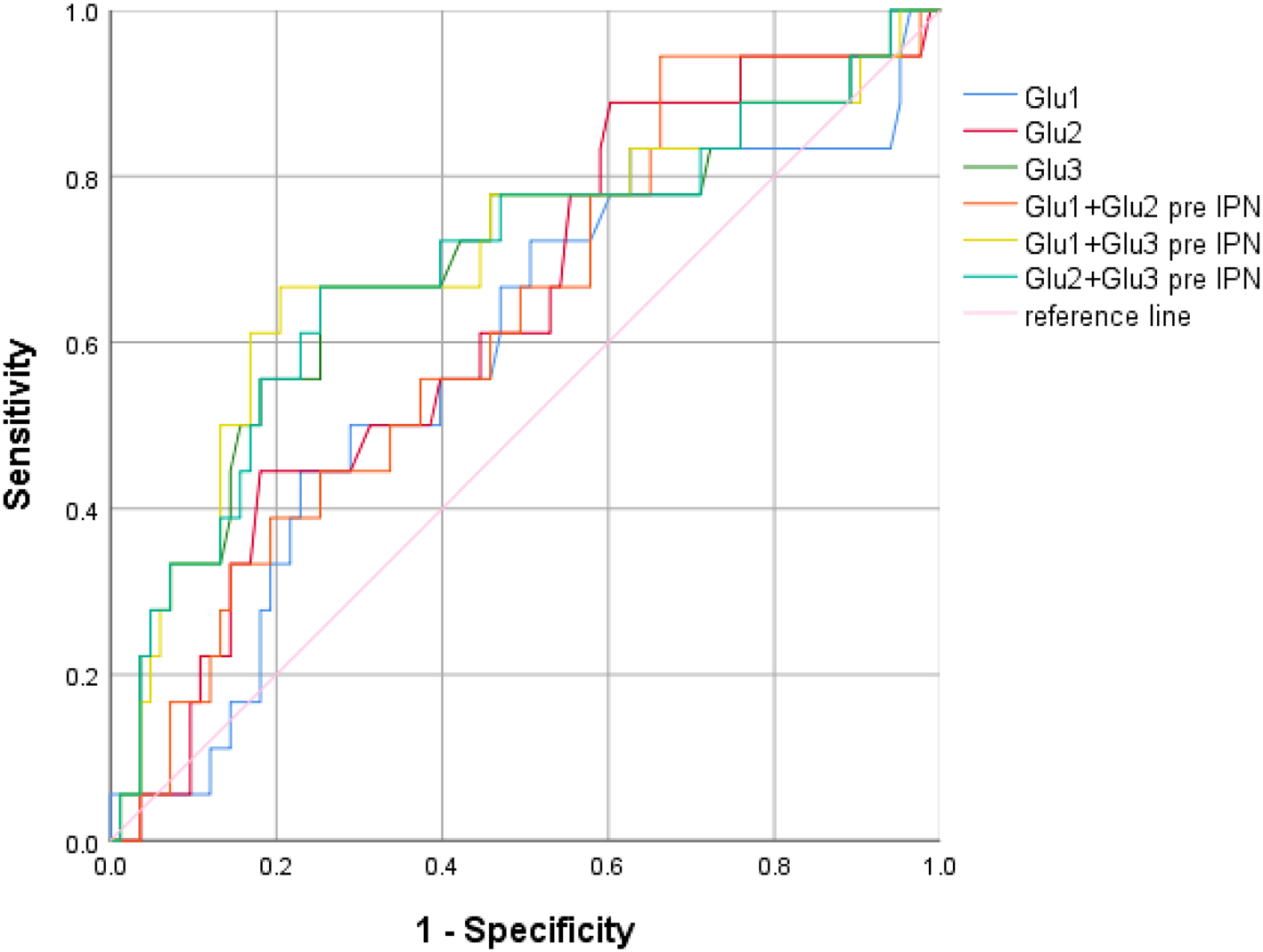
The ROC curve of maximum blood glucose at different periods alone or in combination to predict abdominal infection in critically ill patients with AP.

The ROC curve was used to evaluate the predictive efficacy of maximum blood glucose at different times on IPN in critically ill patients with AP. According to Fig. 4 and Table 9, only Glu3 had the predictive value (AUC: 0.692, *P* = 0.011) and the cutoff value was 18.10 mmol/L. The AUCs of Glu1 + Glu3 and Glu 2 + Glu3 were 0.703 and 0.593, respectively.

**Table 9:** The predictive value of maximum blood glucose for IPN during ICU stay.

Cause	AUC	*P*	95%CI	Youden index	Cut-off Value (mmol/L)	Sensitivity(%)	Specificity (%)
Gallstone							
Glu1,mmol/L	0.336	0.254	0.078–0.594	0.04	8.15	1.00	0.04
Glu2,mmol/L	0.692	0.182	0.489–0.895	0.48	11.95	1.00	0.48
Glu3,mmol/L	0.608	0.452	0.257–0.959	0.40	18.10	0.60	0.80
Glu1 + Glu2	0.6	0.487	0.373–0.827	0.40	0.14	1.00	0.40
Glu1 + Glu3	0.624	0.388	0.266–0.982	0.48	0.21	0.60	0.88
Glu2 + Glu3	0.608	0.452	0.257–0.959	0.4	0.21	0.60	0.80
Hypertriglyceridemia							
Glu1,mmol/L	0.714	0.047	0.523–0.905	0.53	16.35	0.78	0.75
Glu2,mmol/L	0.644	0.179	0.406–0.882	0.43	16.90	0.56	0.88
Glu3,mmol/L	0.656	0.148	0.446–0.866	0.39	17.80	0.67	0.73
Glu1 + Glu2	0.719	0.041	0.516–0.923	0.47	0.22	0.33	0.80
Glu1 + Glu3	0.733	0.03	0.539–0.927	0.59	0.20	0.89	0.7
Glu2 + Glu3	0.672	0.109	0.457–0.887	0.37	0.21	0.67	0.70
All cause							
Glu1,mmol/L	0.585	0.258	0.436–0.734	0.216	13.60	0.722	0.494
Glu2,mmol/L	0.63	0.086	0.492–0.767	0.286	11.95	0.889	0.398
Glu3,mmol/L	0.692	0.011	0.543–0.842	0.414	18.10	0.667	0.747
Glu1 + Glu2	0.622	0.104	0.485–0.760	0.282	0.14	0.944	0.337
Glu1 + Glu3	0.703	0.007	0.553–0.852	0.462	0.21	0.667	0.795
Glu2 + Glu3	0.693	0.011	0.543–0.842	0.414	0.21	0.667	0.747

Glu 1: maximum blood glucose value on day 1; Glu 2: maximum blood glucose value on day 2; Glu 3: maximum blood glucose value on day 3–5.

### Compare differences in etiology while glucose predicting infection

After grouping according to etiology (Tables 7, 8, 9), we performed ROC analysis and found that when blood glucose predicts EPI in patients with biliary acute pancreatitis, the predictive power of Glu3 is higher than that of single or combined blood glucose indicators, and the cut-off value is 14.73 mmol/L, while the etiology is hyperlipidemia, the predictive power of Glu2 is the highest and the cut-off value is 12.25 mmol/L. When blood glucose predicts abdominal infection in patients with hyperlipidemic acute pancreatitis, Glu3 has a higher predictive performance than others, while the cause is biliary, blood glucose cannot predict the occurrence of abdominal infection. When blood glucose predicts IPN in patients with hyperlipidemic acute pancreatitis, Glu1 has a higher predictive performance than others, while the cause is biliary, blood glucose cannot predict IPN.

### The correlation of Glu1 with other variables

The results of rank correlation analysis showed that there was no correlation between Glu1 and gender, etiology, Leucocyte, BUN, ALB, CRP > 150 mg/L, BISAP score, and SIRS at admission. Glu1 was significantly correlated with age, diabetes, HbA1c and PCT (r = 0.285, *P* = 0.003; r = 0.565, *P* = 0.001; r = 0.533, *P* = 0.001; r = 0.396,* P* = 0.001; respectively).

### Association between Glu 1 and outcomes

Divide admission blood glucose levels (Glu 1) into three groups according to the quartile: group A: less than the first quartile (≤ 11.0), group B: between the first and third quartiles (11.0–17.4), and group C: more than the third quartile (> 17.4). Table 10 showed that Glu1 could not influence infection or prognosis.

**Table 10:** Compare whether Glu 1 could influence infection or prognosis.

	Group A	Group B	Group C	*P* value
EPI	10 (38.5%)	31 (62.0%)	15 (57.7%)	0.140
IPN	3 (11.5%)	8 (16.0%)	7 (26.9%)	0.390
Abdominal infection	6 (23.1%)	22 (44.0%)	11 (42.3%)	0.181
Organ failures	15 (57.7%)	35 (70.0%)	20 (76.9%)	0.314
In-ICU death	4 (15.4%)	7 (14.0%)	3 (11.5%)	1.000

Group A: Glu1 ≤ 11.0 mmol/L; Group B: 11.0 mmol/L < Glu1 < 17.4 mmol/L; Group C: Glu1 ≥ 17.4 mmol/L; EPI: extrapancreatic infection; IPN: infected pancreatic necrosis; ICU: intensive care unit.

## Discussion

In this study, the maximum blood glucose was selected as an exposure factor and used to intervene blood glucose control levels in critically ill patients with AP at an appropriate time to avoid infection. We found that the maximum blood glucose on Day2-5 after admission to the ICU can predict infection in critically ill patients with AP. There are differences in etiology while glucose predicting infection. In addition, compared with a single indicator, combined blood glucose values can sometimes increase predictive power.

When people suffer from a serious illness or injury, the body secretes a lot of stress hormones that cause blood glucose levels to rise and many pancreatic islets are damaged in critically ill patients with acute pancreatitis, resulting in more unstable blood glucose levels^12,13^. Our study presented that only 32% patients had previous history of diabetes, but the maximum blood glucose value on day 1 was 14.4 (11.0, 17.4) mmol/L and even higher on later days, indicating that stress hyperglycemia might occur in most patients. Experimental and clinical evidence suggests that stress hyperglycemia can lead to intracellular glucose overload and acute glucotoxicity, leading to oxidative stress, inflammation, endothelial dysfunction, coagulation, osmotic diuresis, inhibition of vasodilation, and impaired ischemic preconditioning. These effects increase organ failure, shock, infection, and mortality. Previous studies have reported a significant association between stress hyperglycemia and clinical outcomes of AP^13^ and infection is easy to occur in the course of acute pancreatitis and is one of the important reasons for the aggravation of the disease and even death^14–16^.

The source of infection mostly comes from the intestinal tract, and the pathogenesis may be: intestinal ischemia and other reasons leading to impaired intestinal barrier function, intestinal bacteria or their endotoxins pass through the intestinal mucosal barrier and enter the mesenteric lymph nodes and portal system, and then invade the systemic circulation and extra-intestinal organ. Some severely infected patients may experience systemic deterioration, secondary to multiple organ dysfunction syndrome (MODS), and eventually death. However, studies have shown that half of the bacterial culture results are not derived from gut flora. Previous authors reported that patients with IPN had a higher incidence of pulmonary infection and bacteremia, which often preceded the diagnosis of IPN^17^. EPI may occur before IPN, so it is particularly important to estimate the risk of infection early.

No relevant study has explored the association between the early blood glucose maximum and the occurrence of EPI and IPN in critically ill patients with AP and there is no universally accepted insulin therapy for blood glucose control. Therefore, the purpose of this study was to explore the predictive value of early blood glucose levels for the occurrence of EPI and IPN in severe AP patients, as well as the level of blood glucose control. In clinical work, dynamic monitoring of leucocytes, CRP, PCT, and other laboratory indicators are often used to assess infection incidence. However, these indicators, including BUN, were not independent risk factors for EPI after inclusion in the multivariate analysis in this study, and could not be used to predict the risk of EPI, which was consistent with a previous study. The study also showed that persistent SIRS in the first week was associated with the occurrence of EPI^7^, and many authors have found that persistent SIRS will affect the prognosis of AP patients^18^. Our study is in accordance with the view that persistent SIRS for more than 48 h will increase the risk of EPI, with statistical significance.

Therefore, it is particularly important to find evaluation indicators that can predict EPI early and intervene in advance^17^. The ideal method should be less invasive, easy to operate, highly accurate, reproducible, and easy to intervene with. Obviously, fingertip blood glucose monitoring meets these requirements.

Though the blood glucose level is influenced by the stress state, if the maximum blood glucose is in the abnormal range, it will have an impact on the patient’s prognosis^19,20^. However, for severe AP patients, there is no recognized blood glucose control value, generally based on the experience of physicians^8,21^.

Although studies have shown that blood glucose-related indicators are associated with in-hospital mortality in critically ill patients with AP, and the maximum blood glucose value has the highest predictive performance^9^. However, the accuracy of the maximum blood glucose value in predicting in-hospital mortality in that study is not high, and blood glucose may increase the risk of infection and then causes death, which potentially could lead to bias. On this basis, our study used the maximum blood glucose value to predict the occurrence of EPI and collected the maximum value in three different periods in the early stage.

The results showed that EPI occurred in 56 patients (54.9%) in this study, which is higher than the incidence in a systematic review of 32% (95% confidence interval 23–41%), the most common being respiratory tract infection (9.2%) and bacteremia (8.4%). According to the analysis, the research subjects included in this study are critically ill patients, so the morbidity may be higher than that of general AP patients, and the retrospective analysis of clinical data is highly subjective due to relying on disease course records. The maximum blood glucose level on the first day after admission to the ICU was not related to the occurrence of EPI in severe AP patients, and the level from the second to the fifth day would influence the occurrence of EPI.

We analyze that patients were at the peak of their stress state on the first day of admission to the ICU, and their blood glucose varied greatly. This exposure factor was no statistical difference in the occurrence of EPI events, so this result appeared. Glu2 and Glu3 had high efficiency in predicting EPI, with AUCs values of 0.805 and 0.782, respectively. Although there is no statistical difference in the accuracy between Glu2 and Glu3 in predicting EPI, the patient’s blood glucose level can be actively intervened early, and the patient’s blood glucose on the second day could be controlled below 12.6 mmol/L to reduce the risk of EPI. The improved accuracy of Glu2 combined with Glu3 in predicting EPI suggests that persistently hyperglycemia will increase the possibility of EPI.

Compare differences in etiology while glucose predicting infection (including EPI, abdominal infection and IPN), we found that in patients with hyperlipidemic acute pancreatitis, the best time to predict the blood glucose level of infection is often earlier than that of biliary AP, which indicating that patients with hypertriglyceridemia AP need to intervene blood glucose levels more actively and earlier, and control it more strictly. We analyze that the mutual promotion of hyperlipidemia and hyperglycemia makes the baseline blood glucose of patients with hypertriglyceridemia AP often higher than that of patients with biliary AP, and patients with biliary AP often have biliary tract infection, so patients with hypertriglyceridemia AP are more likely to occur EPI, abdominal infection and IPN.

In summary, the maximum blood glucose can be used as a predictor of infection in critically ill patients with AP. Patients with hypertriglyceridemia AP need to intervene blood glucose levels more actively and earlier, and control it more strictly.

The significance of this study is that physicians can take an active treatment strategy for patients with suspected infection in the early stage of the course, such as controlling blood glucose levels in a safe range, early blood, sputum, urine, or catheter tip culture, and then adjust the medication according to the culture results, enhance the level of care. This study is a cohort study with limitations such as a small sample size, retrospectives, and subjective evaluation of positive events. Therefore, further randomized controlled trials are needed to evaluate whether actively controlling blood glucose below 12.6 mmol/L can reduce EPI incidence and draw more realistic and effective conclusions, and provide a better basis for clinicians to guide AP patients to control the appropriate blood glucose range. If possible, the type, strain, and time of EPI can be further explored to provide more clues for clinicians to treat infection in the course of AP.

## Data Availability

The datasets used and/or analyzed during the current study are available from the corresponding author on request.
